# [Bis(diphenyl­phosphino)methane-κ^2^ *P*,*P*′][bis­(diphenyl­phosphinometh­yl)diethoxy­silane-κ^2^ *P*,*P*′]bis­(dinitro­gen)­molybdenum(0) benzene 0.7-solvate

**DOI:** 10.1107/S1600536808032364

**Published:** 2008-10-11

**Authors:** Kristina Klatt, Christian Näther, Felix Tuczek

**Affiliations:** a Institut für Anorganische Chemie, Christian-Albrechts-Universität Kiel, Olshausenstrasse 40, D-24098 Kiel, Germany

## Abstract

In the crystal structure of the title compound, [Mo(C_25_H_22_P_2_)(C_30_H_34_O_2_P_2_Si)(N_2_)_2_]·0.7C_6_H_6_, the Mo atoms are coordinated by four P atoms and two N atoms in a distorted octa­hedral mode. The two C atoms of one of the two eth­oxy groups are disordered and were refined using a split model and site-occupation factors of 0.7:0.3. The crystal structure contains a benzene solvent mol­ecule with a site occupation of 70%.

## Related literature

For the coordination chemistry of dinitro­gen, see: MacKay & Fryzuk (2004 ▶). For the synthesis of the ligand, see: Bogza *et al.* (2005 ▶); Leigh & Pickett (1977 ▶).
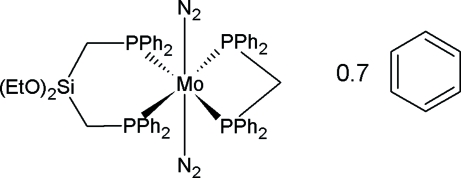


## Experimental

### 

#### Crystal data

[Mo(C_25_H_22_P_2_)(C_30_H_34_O_2_P_2_Si)(N_2_)_2_]·0.7C_6_H_6_
*M* *_r_* = 1107.62
Monoclinic, 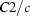

*a* = 26.3512 (19) Å
*b* = 18.2414 (8) Å
*c* = 24.3498 (15) Å
β = 96.114 (8)°
*V* = 11638.0 (12) Å^3^
*Z* = 8
Mo *K*α radiation
μ = 0.40 mm^−1^
*T* = 220 (2) K
0.12 × 0.10 × 0.06 mm

#### Data collection

Stoe IPDS-I diffractometer
Absorption correction: numerical (*X-SHAPE*; Stoe & Cie, 1998 ▶) *T*_min_ = 0.864, *T*_max_ = 0.966
45011 measured reflections
11012 independent reflections
8579 reflections with *I* > 2σ(*I*)
*R*_int_ = 0.074

#### Refinement

*R*[*F* ^2^ > 2σ(*F* ^2^)] = 0.060
*wR*(*F* ^2^) = 0.144
*S* = 1.09
11012 reflections
667 parameters
20 restraints
H-atom parameters constrained
Δρ_max_ = 0.86 e Å^−3^
Δρ_min_ = −0.71 e Å^−3^

### 

Data collection: *IPDS Program Package* (Stoe & Cie, 1998 ▶); cell refinement: *IPDS Program Package*; data reduction: *IPDS Program Package*; program(s) used to solve structure: *SHELXS97* (Sheldrick, 2008 ▶); program(s) used to refine structure: *SHELXL97* (Sheldrick, 2008 ▶); molecular graphics: *XP* in *SHELXTL* (Sheldrick, 2008 ▶); software used to prepare material for publication: *CIFTAB* in *SHELXTL*.

## Supplementary Material

Crystal structure: contains datablocks I, global. DOI: 10.1107/S1600536808032364/bt2807sup1.cif

Structure factors: contains datablocks I. DOI: 10.1107/S1600536808032364/bt2807Isup2.hkl

Additional supplementary materials:  crystallographic information; 3D view; checkCIF report

## Figures and Tables

**Fig. 1.**
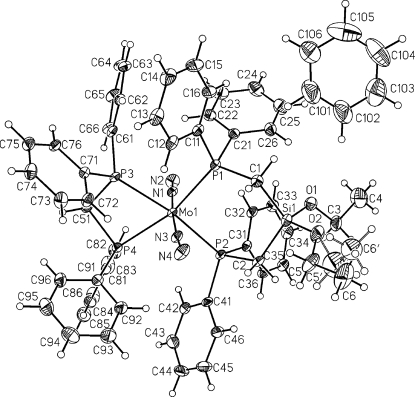
: Crystal structure of the title compound with labelling and displacement ellipsoids drawn at the 30% probability level. The disorder of the carbon atoms is shown with full and open bonds.

## supplementary crystallographic information

### Comment

The structure determination of this compound was undertaken as part of a project on grafting molybdenum(0) dinitrogen complexes to silica, in analogy to studies of Bluemel *et al.* on the immobilization of palladium phosphine complexes to silica for applications in catalysis (Bogza *et al.*, 2005). Later these studies will be extended to semiconducting oxides like SnO_2_. In contrast to Leigh & Pickett who have attached Mo(0) dinitrogen complexes to SnO_2_*via* an axial nitrile ligand (Leigh & Pickett, 1977). the title complex would allow this attachment *via* the equatorial bis(diphenylphosphinomethyl)-diethoxysilane ligand.

In the crystal structure of the title compound, C_55_H_56_MoN_4_O_2_P_4_Si ^.^ (C_6_H_6_)_0.7_ the Mo atoms are coordinated by each two P atoms of the dppm (dppm = diphenylphosphinomethane) and the (Ph_2_PCH_2_)_2_Si(OEt)_2_ ligand as well as two N atoms of two crystallographically independent N atoms of the dinitrogen ligand within distorted octahedra (Tab. 1 and Fig. 1).

### Experimental

A suspension of 281 mg (0.44 mmol) MoCl_4_(dppm) and 260 mg (0.50 mmol) (Ph_2_PCH_2_)_2_Si(OEt)_2_ in 20 ml THF was added to sodium amalgam (200 mg Na, 30.0 g H g)and stirred for 3 h at 0 °C and 16 h at ambient temperature under N_2_. The solution was decanted, filtered and reduced *in vacuo* to 6 ml. 6 ml of methanol were added and the solvent was reduced *in vacuo* again. After addition of another 6 ml of methanol the formed precipitate was filtered off, washed four times with 4 ml of methanol and dried *in vacuo*.

### Refinement

All H atoms were positioned with idealized geometry and were refined with *U*_eq_(H) = 1.2 *U*_eq_ of the parent atom (1.5 for methyl H atoms) using a riding model with C—H = 0.94–0.98 Å. The two carbon atoms of one of the two ethoxy groups are disordered and were refined using a split model. The site occupation factors   refined to 0.7 and 0.3 but were fixed in the final refinement. The atoms on the site of lower occupancy were refined only isotropically. The positions of the benzene molecule are clearly not fully occupied. In the beginning the site occupation factors were refined but later, they were fixed at 0.7. Equivalent bond distances of the disordered ethoxy group and the bond distances in the solvent benzene ring were restrained to be equal with an effective standard deviation 0.02. In addition, the six atoms of the benzene ring were restrained to lie in a common plane.

### Crystal data

**Table:**

[Mo(C_25_H_22_P_2_)(C_30_H_34_O_2_P_2_Si)(N_2_)_2_]·0.7C_6_H_6_	*F*(000) = 4603
*M**_r_* = 1107.62	*D*_x_ = 1.264 Mg m^−^^3^
Monoclinic, *C*2/*c*	Mo *K*α radiation, λ = 0.71073 Å
*a* = 26.3512 (19) Å	Cell parameters from 8000 reflections
*b* = 18.2414 (8) Å	θ = 2–26°
*c* = 24.3498 (15) Å	µ = 0.40 mm^−^^1^
β = 96.114 (8)°	*T* = 220 K
*V* = 11638.0 (12) Å^3^	Block, red
*Z* = 8	0.12 × 0.10 × 0.06 mm

### Data collection

**Table:**

Stoe IPDS-I diffractometer	11012 independent reflections
Radiation source: fine-focus sealed tube	8579 reflections with *I* > 2σ(*I*)
graphite	*R*_int_ = 0.074
φ scans	θ_max_ = 26.0°, θ_min_ = 2.4°
Absorption correction: numerical (X-SHAPE; Stoe & Cie, 1998)	*h* = −32→32
*T*_min_ = 0.864, *T*_max_ = 0.966	*k* = −22→22
45011 measured reflections	*l* = −29→29

### Refinement

**Table:**

Refinement on *F*^2^	Secondary atom site location: difference Fourier map
Least-squares matrix: full	Hydrogen site location: inferred from neighbouring sites
*R*[*F*^2^ > 2σ(*F*^2^)] = 0.060	H-atom parameters constrained
*wR*(*F*^2^) = 0.144	*w* = 1/[σ^2^(*F*_o_^2^) + (0.0347*P*)^2^ + 93.4691*P*] where *P* = (*F*_o_^2^ + 2*F*_c_^2^)/3
*S* = 1.09	(Δ/σ)_max_ = 0.001
11012 reflections	Δρ_max_ = 0.86 e Å^−^^3^
667 parameters	Δρ_min_ = −0.71 e Å^−^^3^
20 restraints	Extinction correction: SHELXL97 (Sheldrick, 2008), Fc^*^=kFc[1+0.001xFc^2^λ^3^/sin(2θ)]^-1/4^
Primary atom site location: structure-invariant direct methods	Extinction coefficient: 0.00028 (7)

### Special details

**Table:**

Geometry. All e.s.d.'s (except the e.s.d. in the dihedral angle between two l.s. planes) are estimated using the full covariance matrix. The cell e.s.d.'s are taken into account individually in the estimation of e.s.d.'s in distances, angles and torsion angles; correlations between e.s.d.'s in cell parameters are only used when they are defined by crystal symmetry. An approximate (isotropic) treatment of cell e.s.d.'s is used for estimating e.s.d.'s involving l.s. planes.
Refinement. Refinement of *F*^2^ against ALL reflections. The weighted *R*-factor *wR* and goodness of fit *S* are based on *F*^2^, conventional *R*-factors *R* are based on *F*, with *F* set to zero for negative *F*^2^. The threshold expression of *F*^2^ > σ(*F*^2^) is used only for calculating *R*-factors(gt) *etc*. and is not relevant to the choice of reflections for refinement. *R*-factors based on *F*^2^ are statistically about twice as large as those based on *F*, and *R*- factors based on ALL data will be even larger.

### Fractional atomic coordinates and isotropic or equivalent isotropic displacement parameters (Å^2^)

**Table:**

	*x*	*y*	*z*	*U*_iso_*/*U*_eq_	Occ. (<1)
Mo1	0.207010 (13)	0.503577 (18)	0.586610 (14)	0.01918 (12)	
N1	0.20784 (14)	0.3935 (2)	0.57550 (15)	0.0266 (8)	
N2	0.20844 (17)	0.3324 (2)	0.56985 (19)	0.0409 (10)	
N3	0.20929 (15)	0.6111 (2)	0.60433 (15)	0.0281 (8)	
N4	0.21203 (19)	0.6703 (2)	0.61769 (18)	0.0435 (11)	
C1	0.33665 (18)	0.5687 (2)	0.62864 (19)	0.0307 (10)	
H1A	0.3279	0.6202	0.6211	0.037*	
H1B	0.3724	0.5627	0.6218	0.037*	
P1	0.29814 (4)	0.51381 (6)	0.57620 (5)	0.0236 (2)	
C2	0.26745 (18)	0.5533 (2)	0.72242 (18)	0.0292 (10)	
H2A	0.2689	0.5438	0.7622	0.035*	
H2B	0.2536	0.6029	0.7161	0.035*	
P2	0.22134 (4)	0.48876 (6)	0.68709 (4)	0.0231 (2)	
O1	0.36655 (13)	0.48060 (19)	0.72215 (14)	0.0403 (8)	
C3	0.3749 (2)	0.4511 (3)	0.7766 (2)	0.0476 (14)	
H3A	0.3443	0.4250	0.7855	0.057*	
H3B	0.3819	0.4909	0.8034	0.057*	
C4	0.4189 (3)	0.4000 (5)	0.7801 (4)	0.088 (3)	
H4A	0.4245	0.3799	0.8171	0.132*	
H4B	0.4491	0.4262	0.7717	0.132*	
H4C	0.4117	0.3606	0.7537	0.132*	
O2	0.36327 (16)	0.6223 (2)	0.73739 (17)	0.0531 (11)	
C5	0.3407 (4)	0.6891 (4)	0.7495 (4)	0.054 (2)	0.70
H5A	0.3096	0.6781	0.7666	0.065*	0.70
H5B	0.3303	0.7139	0.7144	0.065*	0.70
C6	0.3701 (6)	0.7400 (6)	0.7846 (6)	0.095 (5)	0.70
H6A	0.3499	0.7836	0.7892	0.143*	0.70
H6B	0.4006	0.7533	0.7679	0.143*	0.70
H6C	0.3796	0.7175	0.8203	0.143*	0.70
C5'	0.3630 (16)	0.6733 (17)	0.7787 (13)	0.134 (15)*	0.30
H5C	0.3746	0.6515	0.8147	0.161*	0.30
H5D	0.3287	0.6933	0.7801	0.161*	0.30
C6'	0.3982 (12)	0.7304 (17)	0.7648 (15)	0.087 (10)*	0.30
H6D	0.3981	0.7702	0.7912	0.130*	0.30
H6E	0.3874	0.7488	0.7280	0.130*	0.30
H6F	0.4323	0.7103	0.7658	0.130*	0.30
Si1	0.33400 (5)	0.55386 (7)	0.70401 (5)	0.0304 (3)	
C11	0.31535 (17)	0.5657 (2)	0.51541 (19)	0.0291 (10)	
C12	0.28563 (19)	0.6258 (2)	0.49781 (19)	0.0333 (10)	
H12	0.2574	0.6383	0.5165	0.040*	
C13	0.2968 (2)	0.6678 (3)	0.4532 (2)	0.0422 (13)	
H13	0.2762	0.7083	0.4419	0.051*	
C14	0.3382 (2)	0.6502 (3)	0.4252 (2)	0.0530 (16)	
H14	0.3458	0.6785	0.3949	0.064*	
C15	0.3680 (2)	0.5910 (4)	0.4422 (3)	0.0596 (17)	
H15	0.3961	0.5789	0.4232	0.072*	
C16	0.3571 (2)	0.5483 (3)	0.4872 (2)	0.0439 (13)	
H16	0.3779	0.5079	0.4983	0.053*	
C21	0.33546 (17)	0.4297 (2)	0.56971 (19)	0.0292 (10)	
C22	0.31851 (19)	0.3805 (3)	0.5279 (2)	0.0350 (11)	
H22	0.2888	0.3915	0.5045	0.042*	
C23	0.3442 (2)	0.3162 (3)	0.5200 (3)	0.0484 (14)	
H23	0.3327	0.2848	0.4907	0.058*	
C24	0.3870 (2)	0.2981 (3)	0.5553 (3)	0.0576 (17)	
H24	0.4043	0.2539	0.5505	0.069*	
C25	0.4040 (2)	0.3454 (4)	0.5973 (3)	0.0605 (17)	
H25	0.4332	0.3333	0.6213	0.073*	
C26	0.3785 (2)	0.4110 (3)	0.6048 (2)	0.0439 (13)	
H26	0.3906	0.4428	0.6336	0.053*	
C31	0.24594 (17)	0.4021 (2)	0.71857 (18)	0.0265 (9)	
C32	0.27744 (18)	0.3571 (2)	0.69038 (19)	0.0309 (10)	
H32	0.2837	0.3693	0.6542	0.037*	
C33	0.2996 (2)	0.2948 (3)	0.7148 (2)	0.0381 (12)	
H33	0.3209	0.2655	0.6953	0.046*	
C34	0.2904 (2)	0.2757 (3)	0.7676 (2)	0.0391 (12)	
H34	0.3054	0.2331	0.7840	0.047*	
C35	0.25930 (19)	0.3190 (3)	0.7965 (2)	0.0372 (11)	
H35	0.2530	0.3057	0.8325	0.045*	
C36	0.23744 (18)	0.3820 (3)	0.77250 (19)	0.0312 (10)	
H36	0.2167	0.4115	0.7925	0.037*	
C41	0.16564 (17)	0.5045 (3)	0.72604 (17)	0.0294 (9)	
C42	0.12474 (18)	0.4561 (3)	0.7184 (2)	0.0356 (11)	
H42	0.1268	0.4150	0.6955	0.043*	
C43	0.0808 (2)	0.4678 (4)	0.7441 (2)	0.0500 (14)	
H43	0.0538	0.4339	0.7389	0.060*	
C44	0.0762 (2)	0.5281 (4)	0.7768 (2)	0.0552 (16)	
H44	0.0460	0.5365	0.7931	0.066*	
C45	0.1165 (2)	0.5762 (3)	0.7855 (2)	0.0514 (15)	
H45	0.1139	0.6172	0.8085	0.062*	
C46	0.1610 (2)	0.5647 (3)	0.7607 (2)	0.0390 (12)	
H46	0.1882	0.5979	0.7672	0.047*	
P3	0.17645 (4)	0.51372 (6)	0.48719 (4)	0.0226 (2)	
P4	0.11254 (4)	0.50224 (6)	0.56937 (4)	0.0252 (2)	
C51	0.11108 (16)	0.4821 (2)	0.49459 (17)	0.0274 (9)	
H51A	0.1065	0.4298	0.4864	0.033*	
H51B	0.0851	0.5108	0.4721	0.033*	
C61	0.20347 (17)	0.4523 (2)	0.43829 (18)	0.0272 (9)	
C62	0.24413 (17)	0.4761 (3)	0.41067 (18)	0.0302 (10)	
H62	0.2532	0.5260	0.4116	0.036*	
C63	0.2713 (2)	0.4266 (3)	0.3818 (2)	0.0404 (12)	
H63	0.2987	0.4433	0.3634	0.048*	
C64	0.2586 (2)	0.3531 (3)	0.3798 (2)	0.0480 (14)	
H64	0.2773	0.3201	0.3602	0.058*	
C65	0.2184 (2)	0.3286 (3)	0.4065 (2)	0.0467 (14)	
H65	0.2096	0.2787	0.4052	0.056*	
C66	0.1908 (2)	0.3778 (3)	0.4357 (2)	0.0354 (11)	
H66	0.1633	0.3606	0.4537	0.042*	
C71	0.16255 (16)	0.5969 (2)	0.44540 (18)	0.0257 (9)	
C72	0.15542 (19)	0.6634 (3)	0.4722 (2)	0.0348 (11)	
H72	0.1609	0.6658	0.5110	0.042*	
C73	0.1404 (2)	0.7256 (3)	0.4423 (2)	0.0408 (12)	
H73	0.1352	0.7697	0.4608	0.049*	
C74	0.13310 (19)	0.7233 (3)	0.3856 (2)	0.0372 (11)	
H74	0.1233	0.7658	0.3654	0.045*	
C75	0.14024 (19)	0.6582 (3)	0.3584 (2)	0.0351 (11)	
H75	0.1356	0.6566	0.3196	0.042*	
C76	0.15416 (18)	0.5953 (3)	0.38808 (18)	0.0299 (10)	
H76	0.1580	0.5509	0.3693	0.036*	
C81	0.06569 (17)	0.4360 (3)	0.58982 (19)	0.0326 (10)	
C82	0.0772 (2)	0.3616 (3)	0.5871 (2)	0.0448 (13)	
H82	0.1083	0.3471	0.5747	0.054*	
C83	0.0437 (2)	0.3082 (3)	0.6025 (3)	0.0584 (17)	
H83	0.0518	0.2582	0.6001	0.070*	
C84	−0.0016 (2)	0.3297 (4)	0.6215 (3)	0.064 (2)	
H84	−0.0244	0.2941	0.6323	0.077*	
C85	−0.0137 (2)	0.4024 (4)	0.6247 (3)	0.0606 (18)	
H85	−0.0447	0.4163	0.6376	0.073*	
C86	0.01936 (18)	0.4561 (3)	0.6091 (2)	0.0445 (13)	
H86	0.0107	0.5059	0.6115	0.053*	
C91	0.07932 (18)	0.5888 (3)	0.5757 (2)	0.0341 (11)	
C92	0.0885 (2)	0.6268 (3)	0.6251 (2)	0.0465 (14)	
H92	0.1111	0.6068	0.6536	0.056*	
C93	0.0654 (3)	0.6931 (4)	0.6335 (3)	0.0633 (19)	
H93	0.0715	0.7170	0.6678	0.076*	
C94	0.0333 (3)	0.7241 (4)	0.5917 (3)	0.075 (2)	
H94	0.0183	0.7700	0.5968	0.090*	
C95	0.0233 (3)	0.6878 (4)	0.5423 (3)	0.077 (2)	
H95	0.0010	0.7086	0.5137	0.092*	
C96	0.0458 (2)	0.6207 (4)	0.5345 (2)	0.0547 (16)	
H96	0.0383	0.5961	0.5007	0.066*	
C101	0.4698 (5)	0.6214 (8)	0.5784 (6)	0.104 (5)	0.70
H101	0.4360	0.6371	0.5695	0.125*	0.70
C102	0.4863 (4)	0.6103 (7)	0.6313 (6)	0.102 (5)	0.70
H102	0.4639	0.6136	0.6587	0.122*	0.70
C103	0.5348 (5)	0.5946 (7)	0.6440 (7)	0.115 (5)	0.70
H103	0.5472	0.5841	0.6808	0.138*	0.70
C104	0.5668 (5)	0.5936 (8)	0.6041 (8)	0.123 (7)	0.70
H104	0.6020	0.5876	0.6137	0.148*	0.70
C105	0.5483 (6)	0.6012 (8)	0.5501 (7)	0.129 (7)	0.70
H105	0.5704	0.5992	0.5223	0.155*	0.70
C106	0.4987 (5)	0.6113 (10)	0.5373 (7)	0.121 (6)	0.70
H106	0.4843	0.6114	0.5003	0.146*	0.70

### Atomic displacement parameters (Å^2^)

**Table:**

	*U*^11^	*U*^22^	*U*^33^	*U*^12^	*U*^13^	*U*^23^
Mo1	0.02565 (19)	0.01740 (18)	0.01462 (19)	−0.00022 (14)	0.00270 (12)	−0.00092 (12)
N1	0.030 (2)	0.030 (2)	0.020 (2)	−0.0030 (15)	0.0036 (15)	−0.0013 (14)
N2	0.055 (3)	0.0183 (19)	0.049 (3)	−0.0003 (17)	0.003 (2)	−0.0048 (17)
N3	0.040 (2)	0.0262 (19)	0.0179 (19)	0.0019 (16)	0.0007 (16)	0.0026 (14)
N4	0.074 (3)	0.022 (2)	0.033 (2)	−0.0018 (19)	−0.003 (2)	−0.0049 (16)
C1	0.033 (2)	0.029 (2)	0.030 (3)	−0.0038 (18)	0.0031 (19)	0.0028 (18)
P1	0.0267 (5)	0.0229 (5)	0.0210 (6)	−0.0021 (4)	0.0018 (4)	0.0004 (4)
C2	0.042 (3)	0.028 (2)	0.017 (2)	0.0002 (19)	0.0041 (18)	−0.0021 (16)
P2	0.0303 (5)	0.0231 (5)	0.0159 (5)	0.0018 (4)	0.0022 (4)	0.0004 (4)
O1	0.0431 (19)	0.045 (2)	0.033 (2)	0.0044 (16)	0.0015 (15)	0.0067 (15)
C3	0.044 (3)	0.059 (3)	0.036 (3)	0.003 (3)	−0.009 (2)	0.008 (3)
C4	0.084 (6)	0.095 (6)	0.081 (6)	0.037 (5)	−0.007 (4)	0.029 (5)
O2	0.055 (2)	0.049 (2)	0.052 (3)	−0.0136 (19)	−0.0077 (19)	−0.0190 (19)
C5	0.094 (7)	0.030 (4)	0.036 (5)	−0.014 (4)	−0.004 (5)	−0.004 (3)
C6	0.132 (12)	0.045 (6)	0.099 (10)	−0.003 (7)	−0.033 (9)	−0.033 (6)
Si1	0.0339 (7)	0.0310 (6)	0.0252 (7)	−0.0036 (5)	−0.0027 (5)	−0.0028 (5)
C11	0.034 (2)	0.031 (2)	0.021 (2)	−0.0069 (18)	0.0018 (18)	−0.0018 (17)
C12	0.045 (3)	0.028 (2)	0.025 (3)	−0.007 (2)	−0.002 (2)	0.0024 (18)
C13	0.057 (3)	0.033 (3)	0.035 (3)	−0.013 (2)	−0.003 (2)	0.010 (2)
C14	0.060 (4)	0.063 (4)	0.036 (3)	−0.020 (3)	0.007 (3)	0.023 (3)
C15	0.056 (4)	0.086 (5)	0.041 (4)	−0.003 (3)	0.023 (3)	0.018 (3)
C16	0.041 (3)	0.058 (3)	0.034 (3)	−0.001 (2)	0.012 (2)	0.010 (2)
C21	0.030 (2)	0.032 (2)	0.026 (3)	0.0004 (18)	0.0064 (18)	0.0049 (18)
C22	0.037 (3)	0.030 (2)	0.039 (3)	0.0019 (19)	0.008 (2)	−0.003 (2)
C23	0.057 (4)	0.033 (3)	0.057 (4)	0.003 (2)	0.014 (3)	−0.012 (2)
C24	0.052 (4)	0.043 (3)	0.079 (5)	0.019 (3)	0.011 (3)	−0.005 (3)
C25	0.053 (4)	0.061 (4)	0.065 (4)	0.027 (3)	−0.002 (3)	−0.003 (3)
C26	0.038 (3)	0.052 (3)	0.041 (3)	0.012 (2)	−0.003 (2)	−0.006 (2)
C31	0.031 (2)	0.028 (2)	0.020 (2)	−0.0023 (17)	0.0001 (17)	0.0026 (17)
C32	0.041 (3)	0.031 (2)	0.021 (2)	0.0034 (19)	0.0008 (19)	0.0029 (17)
C33	0.047 (3)	0.027 (2)	0.040 (3)	0.008 (2)	0.001 (2)	−0.002 (2)
C34	0.053 (3)	0.028 (2)	0.035 (3)	0.004 (2)	−0.003 (2)	0.011 (2)
C35	0.045 (3)	0.042 (3)	0.024 (3)	0.002 (2)	0.004 (2)	0.014 (2)
C36	0.038 (3)	0.035 (2)	0.021 (2)	0.0049 (19)	0.0057 (19)	0.0072 (18)
C41	0.038 (2)	0.036 (2)	0.015 (2)	0.009 (2)	0.0063 (17)	0.0042 (18)
C42	0.037 (3)	0.046 (3)	0.024 (3)	0.003 (2)	0.001 (2)	0.005 (2)
C43	0.038 (3)	0.072 (4)	0.041 (3)	0.004 (3)	0.007 (2)	0.018 (3)
C44	0.045 (3)	0.084 (5)	0.040 (4)	0.020 (3)	0.020 (3)	0.005 (3)
C45	0.056 (4)	0.060 (4)	0.040 (3)	0.019 (3)	0.017 (3)	−0.005 (3)
C46	0.043 (3)	0.045 (3)	0.030 (3)	0.008 (2)	0.007 (2)	−0.002 (2)
P3	0.0301 (5)	0.0218 (5)	0.0161 (5)	−0.0007 (4)	0.0033 (4)	−0.0019 (4)
P4	0.0267 (5)	0.0284 (5)	0.0205 (6)	−0.0004 (5)	0.0031 (4)	0.0003 (4)
C51	0.029 (2)	0.034 (2)	0.019 (2)	−0.0031 (18)	0.0037 (17)	−0.0031 (17)
C61	0.039 (2)	0.025 (2)	0.017 (2)	0.0018 (18)	−0.0016 (18)	−0.0010 (16)
C62	0.034 (2)	0.035 (2)	0.022 (2)	0.0029 (18)	0.0018 (18)	0.0007 (17)
C63	0.044 (3)	0.053 (3)	0.026 (3)	0.009 (2)	0.009 (2)	−0.004 (2)
C64	0.065 (4)	0.047 (3)	0.034 (3)	0.016 (3)	0.014 (3)	−0.011 (2)
C65	0.076 (4)	0.027 (2)	0.038 (3)	0.005 (2)	0.010 (3)	−0.008 (2)
C66	0.052 (3)	0.030 (2)	0.025 (3)	−0.001 (2)	0.007 (2)	−0.0047 (18)
C71	0.028 (2)	0.027 (2)	0.022 (2)	−0.0008 (17)	0.0009 (17)	0.0008 (17)
C72	0.043 (3)	0.031 (2)	0.028 (3)	0.002 (2)	−0.005 (2)	−0.0050 (19)
C73	0.050 (3)	0.026 (2)	0.044 (3)	0.001 (2)	−0.006 (2)	−0.004 (2)
C74	0.043 (3)	0.030 (2)	0.036 (3)	−0.004 (2)	−0.005 (2)	0.010 (2)
C75	0.039 (3)	0.037 (3)	0.028 (3)	0.000 (2)	0.003 (2)	0.009 (2)
C76	0.037 (2)	0.033 (2)	0.019 (2)	0.0016 (19)	0.0038 (18)	0.0018 (17)
C81	0.029 (2)	0.045 (3)	0.023 (3)	−0.009 (2)	0.0001 (18)	0.0031 (19)
C82	0.035 (3)	0.046 (3)	0.053 (4)	−0.009 (2)	0.003 (2)	0.011 (2)
C83	0.053 (4)	0.049 (3)	0.071 (5)	−0.016 (3)	−0.005 (3)	0.019 (3)
C84	0.043 (3)	0.087 (5)	0.062 (4)	−0.029 (3)	−0.001 (3)	0.026 (4)
C85	0.035 (3)	0.096 (5)	0.051 (4)	−0.017 (3)	0.010 (3)	0.006 (3)
C86	0.029 (3)	0.068 (4)	0.037 (3)	−0.006 (2)	0.007 (2)	0.001 (3)
C91	0.033 (2)	0.043 (3)	0.026 (3)	0.006 (2)	0.0038 (19)	0.002 (2)
C92	0.051 (3)	0.052 (3)	0.035 (3)	0.019 (3)	−0.001 (2)	−0.008 (2)
C93	0.072 (4)	0.061 (4)	0.054 (4)	0.029 (3)	−0.004 (3)	−0.019 (3)
C94	0.079 (5)	0.065 (4)	0.079 (5)	0.044 (4)	0.005 (4)	−0.008 (4)
C95	0.078 (5)	0.086 (5)	0.064 (5)	0.050 (4)	−0.010 (4)	0.001 (4)
C96	0.058 (4)	0.066 (4)	0.037 (3)	0.031 (3)	−0.008 (3)	−0.001 (3)
C101	0.060 (7)	0.139 (13)	0.111 (12)	−0.025 (8)	−0.002 (8)	0.018 (10)
C102	0.058 (7)	0.133 (12)	0.113 (12)	−0.033 (7)	0.004 (7)	0.011 (9)
C103	0.127 (13)	0.102 (11)	0.106 (12)	0.029 (10)	−0.029 (10)	0.029 (9)
C104	0.063 (8)	0.095 (10)	0.21 (2)	0.030 (7)	0.009 (11)	0.033 (13)
C105	0.151 (17)	0.082 (10)	0.172 (19)	0.023 (10)	0.102 (14)	0.015 (11)
C106	0.074 (9)	0.187 (18)	0.104 (12)	−0.027 (10)	0.013 (8)	0.002 (11)

### Geometric parameters (Å, °)

**Table:**

Mo1—N3	2.007 (4)	C41—C42	1.390 (7)
Mo1—N1	2.027 (4)	C41—C46	1.398 (7)
Mo1—P1	2.4485 (11)	C42—C43	1.391 (7)
Mo1—P2	2.4506 (11)	C42—H42	0.9400
Mo1—P3	2.4755 (11)	C43—C44	1.371 (9)
Mo1—P4	2.4801 (11)	C43—H43	0.9400
N1—N2	1.124 (5)	C44—C45	1.376 (9)
N3—N4	1.129 (5)	C44—H44	0.9400
C1—P1	1.840 (5)	C45—C46	1.391 (7)
C1—Si1	1.864 (5)	C45—H45	0.9400
C1—H1A	0.9800	C46—H46	0.9400
C1—H1B	0.9800	P3—C61	1.833 (5)
P1—C21	1.839 (5)	P3—C71	1.842 (4)
P1—C11	1.854 (5)	P3—C51	1.844 (4)
C2—P2	1.839 (4)	P4—C91	1.820 (5)
C2—Si1	1.856 (5)	P4—C81	1.834 (5)
C2—H2A	0.9800	P4—C51	1.854 (4)
C2—H2B	0.9800	C51—H51A	0.9800
P2—C31	1.844 (4)	C51—H51B	0.9800
P2—C41	1.853 (4)	C61—C62	1.394 (6)
O1—C3	1.427 (6)	C61—C66	1.399 (6)
O1—Si1	1.624 (4)	C62—C63	1.389 (7)
C3—C4	1.481 (9)	C62—H62	0.9400
C3—H3A	0.9800	C63—C64	1.381 (8)
C3—H3B	0.9800	C63—H63	0.9400
C4—H4A	0.9700	C64—C65	1.375 (8)
C4—H4B	0.9700	C64—H64	0.9400
C4—H4C	0.9700	C65—C66	1.396 (7)
O2—C5'	1.372 (19)	C65—H65	0.9400
O2—C5	1.401 (9)	C66—H66	0.9400
O2—Si1	1.637 (4)	C71—C76	1.390 (6)
C5—C6	1.431 (11)	C71—C72	1.400 (6)
C5—H5A	0.9800	C72—C73	1.384 (7)
C5—H5B	0.9800	C72—H72	0.9400
C6—H6A	0.9700	C73—C74	1.373 (7)
C6—H6B	0.9700	C73—H73	0.9400
C6—H6C	0.9700	C74—C75	1.383 (7)
C5'—C6'	1.46 (2)	C74—H74	0.9400
C5'—H5C	0.9800	C75—C76	1.385 (6)
C5'—H5D	0.9800	C75—H75	0.9400
C6'—H6D	0.9700	C76—H76	0.9400
C6'—H6E	0.9700	C81—C82	1.394 (7)
C6'—H6F	0.9700	C81—C86	1.403 (7)
C11—C12	1.389 (6)	C82—C83	1.392 (7)
C11—C16	1.396 (7)	C82—H82	0.9400
C12—C13	1.386 (7)	C83—C84	1.384 (10)
C12—H12	0.9400	C83—H83	0.9400
C13—C14	1.383 (8)	C84—C85	1.369 (10)
C13—H13	0.9400	C84—H84	0.9400
C14—C15	1.373 (9)	C85—C86	1.390 (8)
C14—H14	0.9400	C85—H85	0.9400
C15—C16	1.398 (7)	C86—H86	0.9400
C15—H15	0.9400	C91—C92	1.387 (7)
C16—H16	0.9400	C91—C96	1.391 (7)
C21—C26	1.387 (7)	C92—C93	1.379 (8)
C21—C22	1.394 (7)	C92—H92	0.9400
C22—C23	1.378 (7)	C93—C94	1.375 (9)
C22—H22	0.9400	C93—H93	0.9400
C23—C24	1.382 (9)	C94—C95	1.374 (10)
C23—H23	0.9400	C94—H94	0.9400
C24—C25	1.377 (9)	C95—C96	1.381 (8)
C24—H24	0.9400	C95—H95	0.9400
C25—C26	1.394 (8)	C96—H96	0.9400
C25—H25	0.9400	C101—C102	1.330 (12)
C26—H26	0.9400	C101—C106	1.332 (12)
C31—C32	1.399 (6)	C101—H101	0.9400
C31—C36	1.404 (6)	C102—C103	1.315 (12)
C32—C33	1.381 (6)	C102—H102	0.9400
C32—H32	0.9400	C103—C104	1.353 (12)
C33—C34	1.379 (7)	C103—H103	0.9400
C33—H33	0.9400	C104—C105	1.358 (13)
C34—C35	1.384 (7)	C104—H104	0.9400
C34—H34	0.9400	C105—C106	1.323 (13)
C35—C36	1.387 (6)	C105—H105	0.9400
C35—H35	0.9400	C106—H106	0.9400
C36—H36	0.9400		
			
N3—Mo1—N1	174.95 (15)	C35—C34—H34	119.9
N3—Mo1—P1	86.59 (12)	C34—C35—C36	120.0 (5)
N1—Mo1—P1	92.12 (11)	C34—C35—H35	120.0
N3—Mo1—P2	83.99 (11)	C36—C35—H35	120.0
N1—Mo1—P2	91.22 (11)	C35—C36—C31	120.7 (5)
P1—Mo1—P2	93.63 (4)	C35—C36—H36	119.6
N3—Mo1—P3	97.83 (10)	C31—C36—H36	119.6
N1—Mo1—P3	87.16 (10)	C42—C41—C46	117.6 (4)
P1—Mo1—P3	96.52 (4)	C42—C41—P2	118.6 (4)
P2—Mo1—P3	169.78 (4)	C46—C41—P2	123.8 (4)
N3—Mo1—P4	93.01 (11)	C41—C42—C43	120.9 (5)
N1—Mo1—P4	89.57 (11)	C41—C42—H42	119.5
P1—Mo1—P4	163.94 (4)	C43—C42—H42	119.5
P2—Mo1—P4	102.30 (4)	C44—C43—C42	120.8 (6)
P3—Mo1—P4	67.61 (4)	C44—C43—H43	119.6
N2—N1—Mo1	179.3 (4)	C42—C43—H43	119.6
N4—N3—Mo1	175.4 (4)	C43—C44—C45	119.2 (5)
P1—C1—Si1	121.9 (2)	C43—C44—H44	120.4
P1—C1—H1A	106.9	C45—C44—H44	120.4
Si1—C1—H1A	106.9	C44—C45—C46	120.5 (5)
P1—C1—H1B	106.9	C44—C45—H45	119.7
Si1—C1—H1B	106.9	C46—C45—H45	119.7
H1A—C1—H1B	106.7	C45—C46—C41	120.9 (5)
C21—P1—C1	104.7 (2)	C45—C46—H46	119.6
C21—P1—C11	100.3 (2)	C41—C46—H46	119.6
C1—P1—C11	96.5 (2)	C61—P3—C71	102.3 (2)
C21—P1—Mo1	118.99 (15)	C61—P3—C51	107.7 (2)
C1—P1—Mo1	116.36 (16)	C71—P3—C51	100.3 (2)
C11—P1—Mo1	116.60 (15)	C61—P3—Mo1	118.75 (14)
P2—C2—Si1	119.0 (2)	C71—P3—Mo1	128.81 (14)
P2—C2—H2A	107.6	C51—P3—Mo1	95.22 (14)
Si1—C2—H2A	107.6	C91—P4—C81	101.8 (2)
P2—C2—H2B	107.6	C91—P4—C51	107.2 (2)
Si1—C2—H2B	107.6	C81—P4—C51	101.1 (2)
H2A—C2—H2B	107.0	C91—P4—Mo1	117.41 (17)
C2—P2—C31	99.7 (2)	C81—P4—Mo1	130.77 (16)
C2—P2—C41	100.4 (2)	C51—P4—Mo1	94.80 (14)
C31—P2—C41	100.5 (2)	P3—C51—P4	96.4 (2)
C2—P2—Mo1	114.86 (15)	P3—C51—H51A	112.5
C31—P2—Mo1	121.30 (15)	P4—C51—H51A	112.5
C41—P2—Mo1	116.60 (14)	P3—C51—H51B	112.5
C3—O1—Si1	126.0 (3)	P4—C51—H51B	112.5
O1—C3—C4	109.4 (5)	H51A—C51—H51B	110.0
O1—C3—H3A	109.8	C62—C61—C66	118.2 (4)
C4—C3—H3A	109.8	C62—C61—P3	119.6 (3)
O1—C3—H3B	109.8	C66—C61—P3	121.1 (4)
C4—C3—H3B	109.8	C63—C62—C61	120.4 (5)
H3A—C3—H3B	108.2	C63—C62—H62	119.8
C3—C4—H4A	109.5	C61—C62—H62	119.8
C3—C4—H4B	109.5	C64—C63—C62	120.8 (5)
H4A—C4—H4B	109.5	C64—C63—H63	119.6
C3—C4—H4C	109.5	C62—C63—H63	119.6
H4A—C4—H4C	109.5	C65—C64—C63	119.7 (5)
H4B—C4—H4C	109.5	C65—C64—H64	120.2
C5'—O2—C5	38.7 (19)	C63—C64—H64	120.2
C5'—O2—Si1	147.5 (15)	C64—C65—C66	120.1 (5)
C5—O2—Si1	125.5 (5)	C64—C65—H65	119.9
O2—C5—C6	118.4 (9)	C66—C65—H65	119.9
O2—C5—H5A	107.7	C65—C66—C61	120.8 (5)
C6—C5—H5A	107.7	C65—C66—H66	119.6
O2—C5—H5B	107.7	C61—C66—H66	119.6
C6—C5—H5B	107.7	C76—C71—C72	118.2 (4)
H5A—C5—H5B	107.1	C76—C71—P3	122.6 (3)
C5—C6—H6A	109.5	C72—C71—P3	119.0 (3)
C5—C6—H6B	109.5	C73—C72—C71	120.7 (5)
H6A—C6—H6B	109.5	C73—C72—H72	119.7
C5—C6—H6C	109.5	C71—C72—H72	119.7
H6A—C6—H6C	109.5	C74—C73—C72	120.4 (5)
H6B—C6—H6C	109.5	C74—C73—H73	119.8
O2—C5'—C6'	105 (2)	C72—C73—H73	119.8
O2—C5'—H5C	110.7	C73—C74—C75	119.8 (4)
C6'—C5'—H5C	110.7	C73—C74—H74	120.1
O2—C5'—H5D	110.7	C75—C74—H74	120.1
C6'—C5'—H5D	110.7	C74—C75—C76	120.2 (5)
H5C—C5'—H5D	108.8	C74—C75—H75	119.9
C5'—C6'—H6D	109.5	C76—C75—H75	119.9
C5'—C6'—H6E	109.5	C75—C76—C71	120.8 (4)
H6D—C6'—H6E	109.5	C75—C76—H76	119.6
C5'—C6'—H6F	109.5	C71—C76—H76	119.6
H6D—C6'—H6F	109.5	C82—C81—C86	118.2 (5)
H6E—C6'—H6F	109.5	C82—C81—P4	118.2 (4)
O1—Si1—O2	106.6 (2)	C86—C81—P4	123.6 (4)
O1—Si1—C2	114.6 (2)	C83—C82—C81	121.4 (6)
O2—Si1—C2	106.9 (2)	C83—C82—H82	119.3
O1—Si1—C1	108.3 (2)	C81—C82—H82	119.3
O2—Si1—C1	108.3 (2)	C84—C83—C82	119.1 (6)
C2—Si1—C1	112.0 (2)	C84—C83—H83	120.4
C12—C11—C16	118.5 (4)	C82—C83—H83	120.4
C12—C11—P1	118.2 (4)	C85—C84—C83	120.5 (6)
C16—C11—P1	123.4 (4)	C85—C84—H84	119.7
C13—C12—C11	121.2 (5)	C83—C84—H84	119.7
C13—C12—H12	119.4	C84—C85—C86	120.8 (6)
C11—C12—H12	119.4	C84—C85—H85	119.6
C14—C13—C12	120.2 (5)	C86—C85—H85	119.6
C14—C13—H13	119.9	C85—C86—C81	120.0 (6)
C12—C13—H13	119.9	C85—C86—H86	120.0
C15—C14—C13	119.3 (5)	C81—C86—H86	120.0
C15—C14—H14	120.3	C92—C91—C96	117.0 (5)
C13—C14—H14	120.3	C92—C91—P4	117.8 (4)
C14—C15—C16	121.1 (6)	C96—C91—P4	125.2 (4)
C14—C15—H15	119.5	C93—C92—C91	122.0 (5)
C16—C15—H15	119.5	C93—C92—H92	119.0
C11—C16—C15	119.8 (5)	C91—C92—H92	119.0
C11—C16—H16	120.1	C94—C93—C92	119.8 (6)
C15—C16—H16	120.1	C94—C93—H93	120.1
C26—C21—C22	118.0 (4)	C92—C93—H93	120.1
C26—C21—P1	124.2 (4)	C95—C94—C93	119.7 (6)
C22—C21—P1	117.8 (3)	C95—C94—H94	120.1
C23—C22—C21	121.7 (5)	C93—C94—H94	120.1
C23—C22—H22	119.2	C94—C95—C96	120.1 (6)
C21—C22—H22	119.2	C94—C95—H95	120.0
C22—C23—C24	119.9 (5)	C96—C95—H95	120.0
C22—C23—H23	120.1	C95—C96—C91	121.5 (6)
C24—C23—H23	120.1	C95—C96—H96	119.3
C25—C24—C23	119.4 (5)	C91—C96—H96	119.3
C25—C24—H24	120.3	C102—C101—C106	123.5 (14)
C23—C24—H24	120.3	C102—C101—H101	118.2
C24—C25—C26	120.8 (6)	C106—C101—H101	118.2
C24—C25—H25	119.6	C103—C102—C101	118.1 (13)
C26—C25—H25	119.6	C103—C102—H102	121.0
C21—C26—C25	120.3 (5)	C101—C102—H102	121.0
C21—C26—H26	119.9	C102—C103—C104	119.8 (13)
C25—C26—H26	119.9	C102—C103—H103	120.1
C32—C31—C36	117.8 (4)	C104—C103—H103	120.1
C32—C31—P2	120.0 (3)	C103—C104—C105	120.5 (14)
C36—C31—P2	121.9 (3)	C103—C104—H104	119.7
C33—C32—C31	121.1 (5)	C105—C104—H104	119.7
C33—C32—H32	119.4	C106—C105—C104	119.0 (14)
C31—C32—H32	119.4	C106—C105—H105	120.5
C34—C33—C32	120.1 (5)	C104—C105—H105	120.5
C34—C33—H33	119.9	C105—C106—C101	118.1 (14)
C32—C33—H33	119.9	C105—C106—H106	120.9
C33—C34—C35	120.2 (4)	C101—C106—H106	120.9
C33—C34—H34	119.9		
